# Third Generation Antivenomics: Pushing the Limits of the In Vitro Preclinical Assessment of Antivenoms

**DOI:** 10.3390/toxins9050158

**Published:** 2017-05-10

**Authors:** Davinia Pla, Yania Rodríguez, Juan J. Calvete

**Affiliations:** Laboratorio de Venómica Estructural y Funcional, Instituto de Biomedicina de Valencia, CSIC, Jaime Roig 11, 46010 Valencia, Spain; dpla@ibv.csic.es (D.P.); yrodriguez@ibv.csic.es (Y.R.)

**Keywords:** snake venom, antivenom, preclinical assessment of antivenom, third generation antivenomics, size-exclusion analysis of IgG-toxin complexes

## Abstract

Second generation antivenomics is a translational venomics approach designed to complement in vivo preclinical neutralization assays. It provides qualitative and quantitative information on the set of homologous and heterologous venom proteins presenting antivenom-recognized epitopes and those exhibiting impaired immunoreactivity. In a situation of worrying antivenom shortage in many tropical and sub-tropical regions with high snakebite mortality and morbidity rates, such knowledge has the potential to facilitate the optimal deployment of currently existing antivenoms and to aid in the rational design of novel broad specificity antidotes. The aim of the present work was to expand the analytical capability of the immunoaffinity second-generation antivenomics platform, endowing it with the ability to determine the maximal binding capacity of an antivenom toward the different toxins present in a venom, and to quantify the fraction of venom-specific antibodies present in a given antivenom. The application of this new platform, termed third generation (3G) antivenomics, in the preclinical evaluation of antivenoms is illustrated in this paper for the case of antivenom EchiTAb-Plus-ICP^®^ reactivity towards the toxins of homologous (*B. arietans*) and heterologous (*N. melanoleuca*) venoms.

## 1. Introduction

Snakebite envenoming is an occupational and environmental disease that annually kills over 95,000 people residing in some of the world’s most disadvantaged rural communities of tropical and sub-tropical developing countries in sub-Saharan Africa, Asia, Latin America, and parts of Oceania, and that leaves over 300,000 surviving victims with permanent physical disabilities [1,2,3]. The scale of this problem may be far greater than suggested by statistics from hospital-based care. In addition to representing a significant cause of death, snakebite contributes to a depressing cycle of poverty in the world’s most vulnerable communities [4,5]. Despite more than 45 commercial or government antivenom producers existing around the world [6], the shortage of independently validated, demonstrably safe, effective, and affordable snake antivenoms in many affected regions [7,8] is an important contributor to this tropical disease burden [9]. To complicate matters, there is growing evidence suggesting that some marketed antivenoms are clinically ineffective [10,11]. Although necessarily multicomponent in nature, strategies to address this neglect should include the improvement of antivenom availability and the preclinical testing of antivenom efficacy [5,12]. A *sine qua non* requisite for an antivenom entering clinical trials (and eventually being approved for clinical use) is the assessment of its ability to neutralize the most relevant toxic activities of the venoms of the medically relevant snake species within the geographical range where the antivenom is intended to be used [13,14]. To this end, simple experimental protocols have been developed [14,15]. An important progression to prevent the use of poor quality or ineffective antivenoms has been the launch by the WHO of a prequalification scheme of antivenoms for sub-Saharan Africa, a programme also supported by the Global Snakebite Initiative and Médecins Sans Frontières [5,16,17,18].

The shortage of antivenoms can be in part counteracted by designing improved polyspecific antivenoms with a broad neutralizing spectrum, but also by a more rational use of existing antivenoms, i.e., through a systematic and detailed study of their paraspecificity. However, defining the venom cross-reactivity landscape of an antivenom is not a trivial matter, given the well-documented occurrence of venom variability in space (intra- and interpopulation) and time (ontogenetic) within and between all taxonomic levels [19,20,21,22]. This circumstance prevents the use of phylogenetic distance as a measure of venom compositional relatedness, even between closely-related species [23]. To assess the paraspecificity of antivenoms to the level of species-specific toxins, a proteomics-based protocol coined “antivenomics” was introduced in 2008, designed to quantify the extent of cross-reactivity of an antivenom against homologous and heterologous venoms at toxin resolution [24]. The initial protocol, which was based on the in-solution immunoprecipitation of antigen-antibody complexes followed by the chromatographic quantification of the free antigen present in the supenatant, was only suitable for whole IgG antivenoms. This “first generation” approach was subsequently re-designed for the assessment of F(ab’)_2_ and Fab antivenoms [25]. Key to this update was the immobilization of the antivenom molecules on a chromatographic matrix to generate an immunoaffinity column [25]. Among the most relevant advantages of this immunoaffinity-based “second-generation antivenomics” approach, are (i) its ability to yield direct information on both the non-binding and bound toxins and (ii) the smoother baseline of the surrogate reverse-phase chromatograms of the affinity column fractions. These improvements allow better resolution and a more accurate quantification of an antivenom immunorecognition profile than the original immunodepletion protocol. Here, this study reports on a further update of the method that makes it possible to determine the binding capacity of antivenoms for each of a venom’s toxins. In addition, this protocol, called “third generation antivenomics,” includes the quantification of the fraction of antivenom molecules bearing immunoaffinity against venom toxins. This platform has been applied for assessing the immunoreactivity of antivenom EchiTAb-Plus-ICP^®^ (which was used for developing the second generation antivenomics workflow) toward homologous *B. arietans* venom toxins and its paraspecificity against the venom of *N. melanoleuca*.

## 2. Results and Discussion

### 2.1. Toxin-Resolved Immunocapturing Ability of EchiTAb-Plus-ICP^®^ Antivenom towards Homologous Bitis Arietans (Ghana) Venom

**Figure 1:**
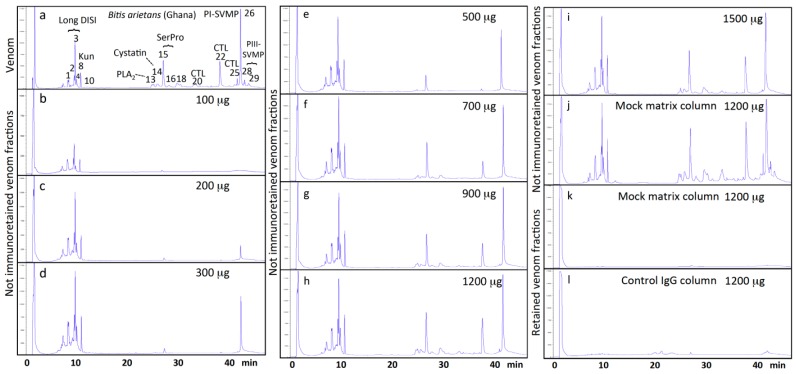
Reverse-phase HPLC profiles of 100 µg of *B. arietans* (Ghana) venom toxins (panel **a**). Toxins eluting in the different chromatographic peaks were reported in [26,27]. DISI, disintegrin; Kun, Kunitz-type inhibitor; PLA_2_, phospholipase A_2_; SerPro, serine proteinase; CTL, C-type lectin-like protein; PI- and PIII-SVMP, snake enom metalloproteinase of class PI and PIII, respectively. Panels **b**–**i**, not immunoretained venom fraction in 300 µL of immobilized (8 mg) EchiTAb-Plus-ICP^®^ antivenom immunoaffinity columns incubated with increasing amounts (100–1500 μg), respectively, of venom proteins. Panels **j** and **k**, not immunoretained and retained venom fractions in a mock column run in parallel to the immunoaffinity columns as matrix control; panel **l**, specificity control, venom proteins retained in a control IgG column.

**Figure 2:**
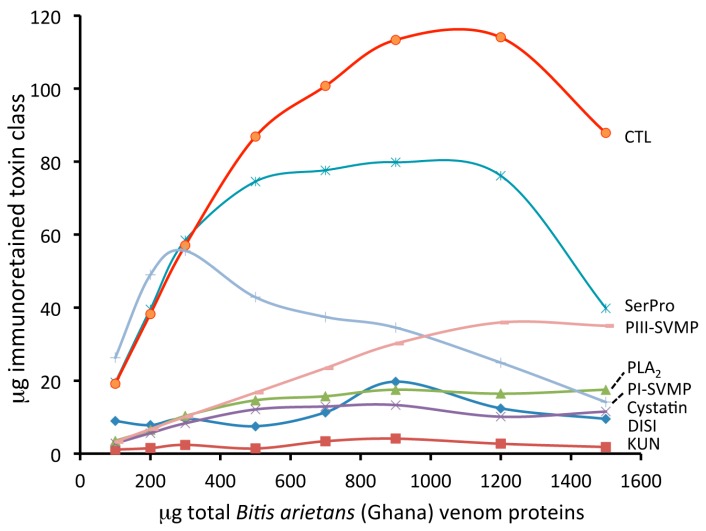
Graphical representation of the binding capacity of the different toxin classes of *B. arietans* (Ghana) venom in 300 µL of immobilized (8 mg) EchiTAb-Plus-ICP^®^ antivenom immunoaffinity columns as a function of the amounts of incubated venom proteins (100–1500 μg). The maximal binding capacity of EchiTAb-Plus-ICP^®^ for toxin “I”, defined as the ratio (µg toxin “i”/mg antivenom) that saturates the affinity column (Rsat“i”), was determined in a series of antivenomic experiments where a set of identical affinity columns are incubated with increasing amounts of venom, and plotting the amount of µg of toxin “i” immunoretained in the columns against the total amount of toxin“i”, (t_“i”_)_TOT_, contained in the incubated venom samples. (t_“i”_)_TOT_ (in µg) = ((% of toxin“i” in the venom) × µg incubated venom)/100, and the relative abundance (%) of toxin “i” corresponds to the % of the total chromatographic area.

Polyclonal antivenoms have different affinities and number of antibodies against the different toxins of a venom. The binding capacity of EchiTAb-Plus-ICP^®^ toward the different toxins present in the venom of *B. arietans* from Ghana was assessed by incubating identical affinity chromatographic columns with increasing amounts of venom until saturation was reached. Comparison of the not immunoretained fractions of a series of eight affinity columns incubated with 100–1500 µg of total venom proteins (Figure 1) showed distinct concentration-dependent patterns of maximal binding for different chromatographic peaks. The calculated venom concentration–dependent immunocapturing capability of the antivenom for each of the chromatographic fractions separated as in panel a of Figure 1 are listed in Supplementary Materials Table S1, and graphically represented in Figure 2. Major PI snake venom metalloproteinase (PI-SVMP) and serine proteinase toxins eluted, respectively, in peaks 26 and 15 (Figure 1, panel a) saturated their affinity binding sites at venom concentration of 300–500 µg. All other venom toxins reached maximal binding at venom concentrations between 900–1200 µg. The maximal binding capacity of the antivenom column was 348.1 µg of total venom proteins, which corresponds to 43.5 mg of *B. arietans* venom proteins per gram of EchiTAb-Plus-ICP^®^ IgG molecules.

Gutiérrez and co-workers [28] have reported an effective dose 50% (ED_50_, µL antivenom/mg venom which reduced by 50% the lethality induced in 18–20 g mice by 5 median lethal doses (LD_50_) of *B. arietans* (Nigeria) venom) for EchiTAb-Plus-ICP^®^ of 181–312 µL. This figure corresponded to a neutralization capacity of 66–114 mg venom proteins per gram of antivenom. In more recent investigations, Segura et al. [29] and Sánchez et al. [30] reported, respectively, ED_50_s for the same antivenom of 42.3–66.6 and 27.5–80 mg venom/g IgG. EchiTAb-Plus-ICP^®^ was able to completely prevent lethality at a higher dose, namely 667 µL antivenom/mg venom [29], at which 1 g of antivenom would neutralized 31 mg of venom proteins. These data agree with those derived in this work for the maximal venom proteins binding capacity of EchiTAb-Plus-ICP^®^ antivenom. A combination of experimental (in solution versus immobilized antivenom) and biological (geographic variability of *B. arietans* venom [31]) particularities may account for the small differences noted.

Although a comparison of the levels of immune recognition gathered from antivenomics with the in vivo neutralization capacity of an antivenom is not straightforward (since both experiments involve radically different protocol, an immunocapturing capability of ~20–25% of total venom proteins correlates with a good outcome in in vivo neutralization tests [32]. In this respect, the results shown in Supplementary Materials Table S1 indicate that, with the sole exception of disintegrins and a Kunitz-type serine proteinase inhibitor, the degree of immunodepletion of all other venom toxins reached 55 to 93% at maximal binding conditions. The good correlation between third generation antivenomics and in vivo neutralization assays highlights the complementarity of both approaches to assess the preclinical efficacy of an antivenom to neutralize venom lethality [30]. In addition, antivenomics has the unique ability of providing the grounds for rationalizing the homologous or paraspecific efficacy of an antivenom, valuable knowledge for aiding in the design of immunogen mixtures for generating antivenoms exhibiting improved therapeutic scope.

### 2.2. Toxin-Resolved Immunocapturing Ability of EchiTAb-Plus-ICP^®^ Antivenom towards Heterologous Forest Cobra (Naja melanoleuca, Ghana) Venom

**Figure 3:**
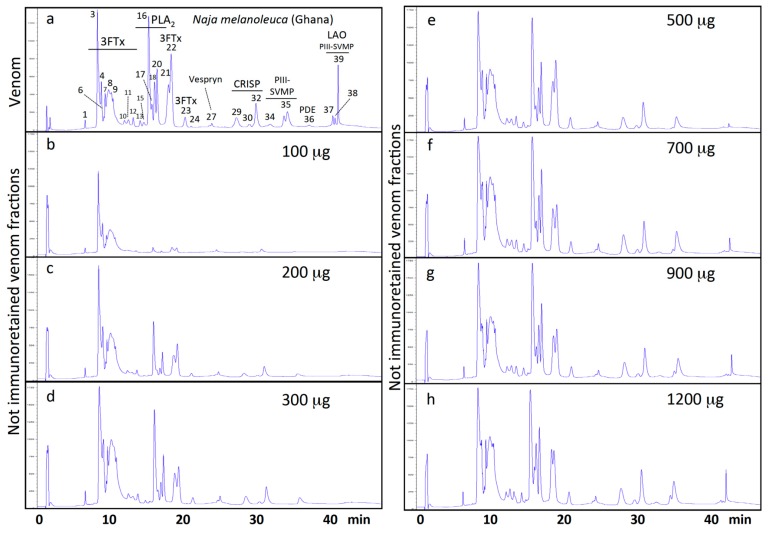
Reverse-phase HPLC profiles of 100 mg of *N. melanoleuca* (Ghana) venom toxins (panel **a**). Toxins eluting in the different chromatographic peaks has been reported in [33] and unpublished results. 3FTx, three-finger toxin; PLA_2_, phospholipase A_2_; CRISP, cysteine-rich secretory protein; PIII-SVMP, snake venom metalloproteinase of class PIII; PDE, phosphodiesterase; LAO, L-amino acid oxidase. Panels **b**–**h**, not immunoretained venom fraction in 300 µL of immobilized (8 mg) EchiTAb-Plus-ICP^®^ antivenom immunoaffinity columns incubated with increasing amounts (100–1200 μg), respectively, of venom proteins.

**Figure 4:**
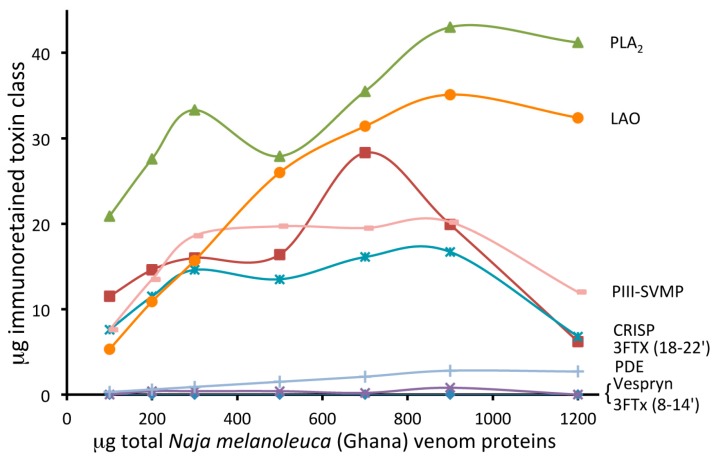
Graphical representation of the binding capacity of the different toxin classes of *N. melanoleuca* (Ghana) venom in 300 µL of immobilized (8 mg) EchiTAb-Plus-ICP^®^ antivenom immunoaffinity columns as a function of the amounts of incubated venom proteins (100–1200 μg). The maximal binding capacity of EchiTAb-Plus-ICP^®^ for forest cobra venom toxins was calculated as described in the legend of Figure 2.

The ability of EchiTAb-Plus-ICP^®^ to immunocapture individual toxins of Ghanan *N. melanoleuca* was assessed as described above for *B. arietans*. To this end, affinity columns containing 8 mg of immobilized antivenom IgG molecules were challenged with 100–1200 µg of forest cobra venom proteins. Antivenom IgGs failed to bind the three-finger toxin (3FTx) molecules eluted between 8–14 min across the entire range of venom concentrations tested (Supplementary Materials Table S2 and Figure 3 and Figure 4). The other venom toxins were recognized by antivenom molecules, albeit saturating their immunorecognition sites at different antivenom:venom ratios and exhibiting disparate degrees of immunocapturing at maximal binding: 3FTxs eluting between 18–22 min and vespryn showed immunoaffinity binding yields of 12–28%; cysteine-rich secretory proteins (CRISP), 26–56%; phospholipases A_2_ (PLA_2_s), 28–63%; PIII-snake venom metalloproteinases (SVMPs), 31–68%; and L-amino acid oxidase (LAO), 61–73%.

### 2.3. Toxicovenomics-Guided Toxin-Resolved Antivenomics Predictions

The maximal binding capacity of the 8 mg EchiTAb-Plus-ICP^®^ antivenom column was 156.5 μg of total venom proteins (Supplementary Materials Table S2). This figure corresponded to 19.6 mg of *N. melanoleuca* venom proteins per gram of IgG molecules. Assuming that the degree of toxin immunorecognition by an antivenom represents a measure of its capability to neutralize the venom’s lethal activity, a 10 mL vial of EchiTAb-Plus-ICP^®^ (40 mg IgG/mL; calculated ED_50_ for *N. melanoleuca* (Uganda) = 0.66 mg/kg (95% confidence interval 0.49–0.92 mg/kg for 18–20 g mice) [33]), could theoretically neutralize 8–16 LD_50_s. Native to the central and western parts of the African continent, the forest cobra represents the largest true cobra (*Naja*) species [34]. It yields 0.5–1.1 g venom proteins per milking [35]. Thus, 51–102 vials of EchiTAb-Plus-ICP^®^ might be required to bind, and eventually neutralize, such an amount of venom proteins. However, the antivenomics data highlight the poor immunorecognition of venom 3FTxs, the most abundant (57% of the venom proteome) and lethal components of forest cobra venom. Most relevant, α-neurotoxins eluting between 8–14 min comprise 24% of *N. melanoleuca* venom toxins, exhibit LD_50_s <0.2 µg/g mouse [33] and show no affinity for EchiTAb-Plus-ICP^®^ antibodies. This means that a bite injecting the whole venom load can inoculate as much as 120–240 mg of 3FTxs toxins, quantities capable of killing 4–8 75 kg humans, and against which the antivenom is not effective. Clearly, clinical studies are required to determine the effectiveness of EchiTAb-Plus-ICP^®^ against envenomings by *N. melanoleuca*. However, toxicovenomics and toxin-resolved antivenomics evidence strongly suggest a poor outcome for this antivenom against forest cobra envenomations.

### 2.4. Quantification of Venom-Specific Antivenom Antibodies

In almost all existing antivenoms, antibodies toward non-venom antigens represent the most abundant molecules. IgGs from normal and hyperimmune sera are indistinguishable chemically and structurally, and quantification of the content of anti-toxin antibodies is seldom determined as part of the preclinical evaluation of an antivenom. However, this parameter is relevant to compare both the effectiveness of antivenoms with the same nominal venom specificity but manufactured by different companies, and the binding capacity of the same antivenom toward homologous and heterologous venoms. Estimation of the relative abundance of therapeutic molecules in an antivenom preparation has been addressed through immunochemical methods, e.g., by determining the ratio of potency (ED_50_ expressed as mg venom neutralized per mL antivenom) to total protein content (mg/mL) [36]. The fraction of therapeutic molecules present in the antivenom preparation ([Ab_ther_]) can be calculated using the Equation (Ab_ther_) = (ED_50_/(M_ave_ toxins))/((Ab)/M_Ab_), where (M_ave_ toxins) is the average molecular mass of the venom toxins, (Ab) is the total protein concentration (mg/mL) of the antivenom preparation, and M_Ab_ is the molecular mass of the active antivenom’s molecules (158 kDa for IgG, 100 kDa for (F(ab’)_2_, 50 kDa for Fab). Here, analysis of size-exclusion HPLC (SEC) elution profiles of increasing venom:antivenom ratio mixtures [37,38,39,40] have been applied to quantify stable (EchiTAb-Plus-ICP^®^ antibody-(*B. arietans* toxin)) complexes. SE-HPLC elution profiles are mechanistically easy to interpret and mathematically modeled using dose-response functions based on the law of mass action.

**Figure 5:**
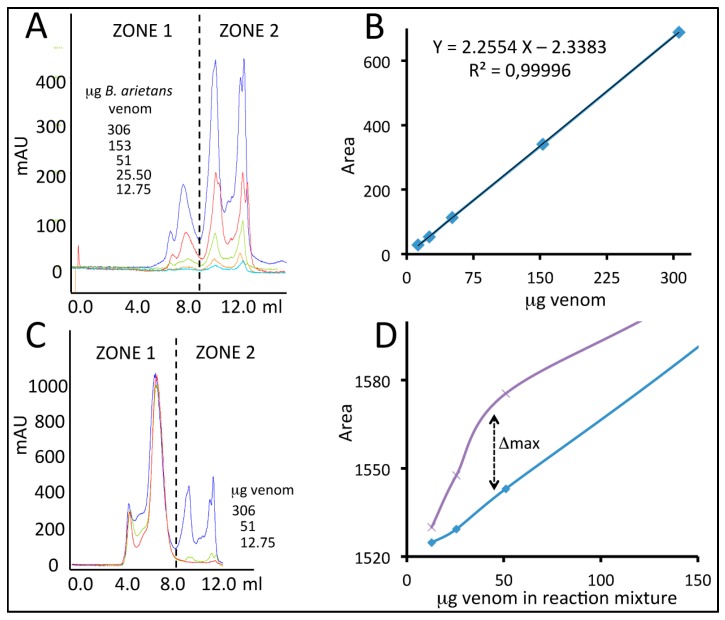
Size-exclusion chromatographic approach used to quantify the fraction of EchiTAb-Plus-ICP^®^ IgG molecules toxin capable of forming stable complexes with *B. arietans* (Ghana) venom toxins. Panel (**A**) displays SEC elution profiles of different amounts of B. arietans venom proteins used to construct the calibration standard curve displayed in panel (**B**). Zone 1 includes venom components that overlap with antivenom IgGs, and antigen-antibody complexes. Zone 2 is the region of the chromatogram where free venom proteins are eluted. Panel (**C**) displays superposition of SEC profiles of reaction mixtures comprising a constant amount of antivenom (300 µg) incubated with increasing amounts (12.75–306 µg) of *B. arietans* venom proteins. Increment of zone 1 area was attributed to antivenom-venom complex formation. and calculated using the Equation (Area venom complexed with antivenom IgG) = (total area zone-1) − (area 300µg IgG) − (area zone-1 control venom curve). Panel (**D**) displays the calculated area attributed to antivenom-venom complexes as a function of the amount of venom in the reaction mixture. Blue line, sum of the zone 1 areas of the standard curves of venom and antivenom; Magenta line, area of zone 1 of the chromatographic profiles of venom-antivenom mixtures. The maximal increment in area was then used to calculate, using the calibration curve plotted in (**B**), the maximal binding capacity of a defined amount of EchiTAb-Plus-ICP^®^ antivenom.

Figure 5 illustrates SEC elution profiles of reaction mixtures of a constant amount of antivenom (300 µg) incubated with increasing amounts (12.75–306 µg) of *B. arietans* venom. The observed profile changes were attributed to antivenom-venom complex formation, resulting in mass transfer from zone 2 (Z2), where only venom protein elute, to zone 1 (Z1), where the elution of some high molecular mass venom proteins, antivenom IgG molecules, and antigen-antibody complexes partially overlap. To quantitate the fraction of the zone 1 area increase corresponding to the formation of venom-antivenom complexes, it was necessary to subtract from each reaction profile the area contributed by the free reactants. To this end calibration curves for free antivenom (AV) IgG and venom (VE) alone were constructed and the amount of venom proteins bound by a constant amount of antivenom at any venom:antivenom ratio was calculated by Equation: (Area AV-VE complexes) = (area Z1 AV-VE chromatogram) − (area control AV) − (area control VE), where the areas of control AV and control VE correspond to those of equal amounts used in the reaction mixture.

Using this approach, it was calculated that 1 gram (6.6 micromoles) of EchiTAb-Plus-ICP^®^ IgGs had maximal binding capacity of 51 mg (2 micromoles) *B. arietans* venom proteins. This value agrees well with the quantification obtained by antivenomics in Section 2.1. Assuming that each IgG molecule bound two molecules of target venom protein, we estimated that 15% of EchiTAb-Plus-ICP^®^ antibody molecules target *B. arietans* (Ghana) venom toxins. Taking into account that EchiTAb-Plus-ICP^®^ is a polyspecific antivenom and that *B. arietans* venom is only one of the three venoms used in its generation [26], this figure is in agreement with the value, frequently quoted in the toxinology community, of 10–40% of non-immunopurified animal antivenom antibodies being directed against venom components [36,41,42].

Attempts to measure the amount of IgGs specific against *N. melanoleuca* toxins gave results not significantly different from the control. This failure in the determination of stable immunocomplexes may be attributed to a combination of low immunoreactivity and low affinity of EchiTAb-Plus^®^ IgGs against the toxins of the forest cobra venom, which was not included in the immunization mixture used to generate the antivenom.

## 3. Concluding Remarks and Perspectives

Antivenomics is translational venomics applied for the functional characterization of antivenoms. In addition to the physicochemical characteristics of the immune molecules (e.g., homogeneity and purity of IgG, F(ab’)_2_, or Fab), relevant functional parameters that should be determined in the preclinical stage of an antivenom include (i) its efficacy to neutralize the toxic effects of the target venom; (ii) toxin-specificity and paraspecificty; and (iii) the quantification of the fraction of therapeutic molecules. Analysis of the neutralizing activity of a venom’s toxic effects, including lethality, in vivo or ex vivo systems are recommended [15,18], while for the molecular aspects of the preclinical assessment (immunoreactivity profiling and quantification of venom-specific molecules), in vitro protocols have been developed [25,40]. The testing of antivenoms on animals raises important ethical considerations, and the WHO recommends the systematic second-generation antivenomics quality control for each new batch of an antivenom before animal experiments are undertaken [18]. As pinpointed in the review by Gutiérrez et al. of this issue [15], in order to reduce the use and suffering of experimental animals, the philosophy of the 3Rs (i.e., Reduce, Refine, and Replace animal tests) needs to be actively promoted in the field of antivenoms, including the systematic use of analgesics and the development of novel in vitro assays in substitution of in vivo tests. To fulfill these recommendations, the second generation antivenomics protocol has been updated to include the determination of the maximal binding capacity of the immunoaffinity column for the different toxins of a venom and the quantification of the fraction of toxin-specific antibodies present in the antivenom. SE-HPLC elution profile analysis has been previously used as a rapid means of separating and quantifying stable antibody (Ab)-antigen (Ag) complexes from unbound reactants [38,39,40]. SE-HPLC has the advantage over other chromatographic methods in that it can be performed under conditions that preserve the native structure and binding characteristics of venoms and antivenoms. This improvement in the analytical capabilities of the protocol termed “third generation (3G) antivenomics” allows a more detailed functional comparison of nominally identical antivenoms in terms of immunological profile. As shown in the case of *N. melanoleuca*, the interpretation of 3G antivenomics in the context of the venom toxicovenomic profile could eventually replace in vivo tests, with the consequent reduction of the use of animals in the evaluation of antivenom protection against venom-induced lethality.

## 4. Materials and Methods

### 4.1. Venoms and Antivenom

Venom from the puff adder, *Bitis arietans*, from Ghana was generously provided by Dr. Robert A. Harrison (Liverpool School of Tropical Medicine, Liverpool, UK), and its toxin composition has been previously characterized [26,27]. Venom from the forest (or black) cobra, *Naja melanoleuca*, from Ghana was purchased from Latoxan (Valence, France). Proteomic characterization of the venom of *N. melanoleuca* from Uganda has been reported [33], and that of the same Ghanan species will be reported elsewhere. Antivenom EchiTAb-Plus-ICP© (batch n°: 5620715PALQ; manufactured: July 2015; expiring date: July 2018) was generously provided by Prof. José María Gutiérrez (Instituto Clodomiro Picado, Universidad de Costa Rica, San José, Costa Rica). Details of its production and immunological profile have been reported [28,29,30,31,43,44,45,46].

### 4.2. Antivenomics

The EchiTAb-Plus-ICP^®^ antivenom (Instituto Clodomiro Picado, Universidad de Costa Rica, San José, Costa Rica) was dialysed against MilliQ^®^ water, lyophilised, and reconstituted in 0.2 M NaHCO_3_, 0.5 M NaCl, pH 8.3 (coupling buffer). Antivenom affinity columns were prepared in batch. 6 mL of CNBr-activated Sepharose™ 4B matrix (Ge Healthcare, Buckinghamshire, UK) packed in a ABT column (Agarose Bead Technologies, Torrejón de Ardoz, Madrid, Spain) and washed with 10 matrix volumes of cold 1 mM HCl, followed by two matrix volumes of coupling buffer to adjust the pH of the column to 7.0–8.0. CNBr-activated instead of *N*-hydroxysuccinimide (NHS)-activated matrix was employed because NHS released during the coupling procedure absorbs strongly at 280 nm, thus interfering with the measurement of the concentration of antibodies remaining in the supernatant of the coupling solution. The concentrations of the antivenom stock solutions were determined spectrophotometrically using an extinction coefficient for a 1 mg/mL concentration (ε_0.1%_) at 280 nm of 1.36 (mg/mL) × cm^−1^ [47]. 200 mg of EchiTAb-Plus-ICP^®^ antivenom were dissolved in the 2× matrix volume of coupling buffer and incubated with the 6 mL matrix column for 4 h at room temperature. Antivenom coupling yield, estimated measuring A_280_ before and after incubation with the matrix and using the Beer-Lambert Law, was 26.7 mg/mL. After the coupling, remaining active groups were blocked with 12mL of 0.1 M Tris-HCl, pH 8.5 at room temperature for 4 h. Seven affinity columns containing 300 µL of immobilized (8 mg) EchiTAb-Plus-ICP^®^ antivenom were alternately washed with three matrix volumes of 0.1 M acetate containing 0.5 M NaCl, pH 4.0–5.0, and three matrix volumes of 0.1 M Tris-HCl, pH 8.5, repeated six times. The columns were then equilibrated with five volumes of working buffer (PBS, 20 mM phosphate buffer, 135 mM NaCl, pH 7.4) and incubated with increasing amounts (100–1200 μg total venom proteins) of *N. melanoleuca* from Ghana dissolved in ½ matrix volume of PBS, and incubated for 1 h at 25°C using an orbital shaker. Another set of eight affinity columns were incubated with increasing amounts (100–1500 μg total proteins) of *B. arietans* venom from Ghana dissolved in ½ matrix volume of PBS, and incubated for 1 h at 25°C using an orbital shaker. As specificity controls, columns of 300 μL of CNBr-activated Sepharose™ 4B matrix, without (mock) or with 8 mg of immobilized control horse IgGs, were incubated with venom and developed in parallel to the immunoaffinity columns.

The non-retained fractions of columns incubated with 100-300 μg, 500-700 μg, 900 μg, 1200 μg, and 1500 μg of venom were recovered with 5×, 10×, 15×, 20×, and 25× matrix volumen of PBS, respectively. Sintiprungat et al. [48] have recently reported that the choice of eluting buffer can significantly alter the binding of the proteins to the matrix and consequently the conclusions drawn from antivenomics studies. In this study, the following elution conditions, alone or combined, were compared: 0.1 M glycine-HCl, pH 2.7; 0.1 M glycine, pH 10.0; 0.1 M glycine-HCl, pH 2.7, including 2M NaCl; 0.1% trifluoroacetic acid (TFA) in 30% acetonitrile (ACN), followed by 0.1% TFA in 70% ACN; 3M sodium thiocyanate (NaSCN > 99.99%) (Sigma-Aldrich Quimica, Tres Cantos, Madrid, Spain); and 0.1 M glycine-HCl, pH 2.7 followed by 3M sodium thiocyanate. Eluates were collected in fractions of ½ column volume, brought to neutral pH and/or desalted, concentrated by Speed-Vac (Savant™, Thermo Scientific Inc., West Palm Beach, FL, USA), and analyzed by SDS-PAGE and reverse-phase HPLC (as below). Best results were obtained with 0.1M glycine-HCl, pH 2.7. Therefore, in all the experiments described in this work, the immunocaptured proteins were eluted with this buffer, the eluates brought to neutral pH with 1M Tris-HCl, pH 9.0, and those fractions containing more than 90-95% of the eluted proteins were pooled for further analysis.

To avoid saturation of the downstream reverse-phase chromatographic analysis, a fraction (1/*x*, *x* = 1, 2, 3, 4, and 5 of the non-retained and the immunocaptured venom fractions recovered, respectively, with 5×, 10×, 15×, 20×, and 25× matrix volumen) were concentrated in a Savant™ SpeedVac™ vacuum system (ThermoFisher Scientific, Waltham, MA, USA) to 40 μL, and fractionated by reverse-phase HPLC using an Agilent LC 1100 High Pressure Gradient System (Santa Clara, CA, USA) equipped with a Discovery^®^ BIO Wide Pore C_18_ (15 cm × 2.1 mm, 3 μm particle size, 300 Å pore size) column (Supelco Sigma-Aldrich, Bellefonte, PA, USA) and a DAD detector (Agilent Technologies, Santa Clara, CA, USA). The column was developed at a flow rate of 0.4 mL/min with a linear gradient of 0.1% TFA in MilliQ^®^ water (Merck-Millipore, Darmstadt, Germany) (solution A) and 0.1% TFA in acetonitrile (solution B), isocratic (5% B) for 1 min, followed by 5–25% B for 5 min, 25–45% B for 35 min, and 45–70% B for 5 min. Eluate was monitored at 215 nm with a reference wavelength of 400 nm. The fraction of non-immunocaptured molecules was estimated as the relative ratio of the chromatographic areas of the toxin recovered in the non-retained (NR) and retained (R) affinity chromatography fractions using the Equation % NR_i_ = 100 − [(R_i_/(R_i_ + NR_i_)) × 100], where R_i_ corresponds to the area of the same protein “i” in the chromatogram of the fraction retained and eluted from the affinity column. However, for some toxins that were poorly recovered in the column-retained fraction owing to the high affinity of the binding, the percentage of non-immunocaptured toxin“i” (% NR_toxin“i”_) was calculated as the ratio between the chromatographic areas of the same peak recovered in the non-retained fraction (NR_toxin“i”_) and in the injected crude reference venom (V_toxin“i”_), using the Equation %NR_toxin“i”_ = (NR_toxin“i”_/V_toxin“i”_) × 100.

### 4.3. Quantification of Antivenom-Toxin Complexes by Size-Exclusion Chromatography

Calibration curves were constructed plotting the chromatographic area as a function of increasing amounts of EchiTAb-Plus-ICP^®^ antivenom (50–750 µg) and total venom proteins of *B. arietans* (12.75, 25.5, 51, 153, 306 µg). To quantify the abundance of toxin-specific IgG molecules in EchiTAb-Plus-ICP^®^ antivenom, constant amounts of 300 µg of antivenom were incubated with the same amounts *B. arietans* total venom proteins used to construct the calibration curves in 50 µL of 0.1 M ammonium acetate, pH 6.9, for 1h at 37 °C. Assuming average molecular masses of 25 kDa for *B. arietans* toxins, these mixtures correspond to venom:antivenom molar ratios of 0.33:1, 0.5:1, 1:1, 3:1, 6:1. Venom-antivenom mixtures were fractionated by size-exclusion chromatography (SEC) using an ETTAN-LC chromatograph (GE Healthcare, Buckinghamshire, UK) and a Bio-Sil SEC 250 (300 × 7.8 mm) column (Bio-Rad Laboratories, Inc., Hercules, CA, USA) equilibrated in the same buffer and calibrated with a mixture of dextran blue (>2 MDa), β-amilase (224 kDa), BSA (66.3 kDa), carbonic anhydrase (29 kDa) and ATP (507 Da). The flow rate was set to 0.7 mL/min, the eluate was monitored at 215 nm and the chromatographic fractions, collected manually, were analyzed for the presence of venom proteins by RP-HPLC using the chromatographic conditions described in Section 4.2.

## Supplementary Materials

The following are available online at www.mdpi.com/2072-6651/9/5/158/s1, Table S1: Total and concentration-dependent immunoretained *B. arietans* (Ghana) venom proteins by EchiTAb-Plus-ICP^®^ antivenom affinity column, Table S2: Total and concentration-dependent immunoretained *N. melanoleuca* (Ghana) venom proteins by EchiTAb-Plus-ICP^®^ antivenom affinity column.
