# Supplementary material for: Third Generation Antivenomics: Pushing the Limits of the In Vitro Preclinical Assessment of Antivenoms
Source: Toxins (Basel). 2017 May 10;9(5):158. doi: 10.3390/toxins9050158 (PMC5450706; doi:10.3390/toxins9050158)
Supplement: Supplementary file 1 [file toxins-09-00158-s001.pdf]

Supplementary Materials: Third Generation Antivenomics: Pushing the Limits of the in Vitro Preclinical Assessment of Antivenoms

Davinia Pla, Yania Rodríguez and Juan J. Calvete

Table S1. Total and concentration-dependent immunoretained (RET) *B. arietans* (Ghana) venom proteins by EchiTAb-Plus-ICP® antivenom affinity column.

		Bitis arietans **(Ghana) total venom proteins g)**								
**RP-HPLC fraction**		**100**	**200**	**300**	**500**	**700**	**900**	**1200**	**1500**	**Toxin class**
**2**	g TOTAL	2,1	4,2	6,3	10,4	14,6	18,8	25,0	31,3	
	g RET	0,6	0,1	0,4	2,2	2,4	**2,7 (14.3%)**	1,8	1,9	
**3**	g TOTAL	9,6	19,3	28,9	48,2	67,5	86,8	115,7	144,6	**DISI**
	g RET	6,3	5,3	6,3	3,0	5,7	**10,9 (12,6%)**	6,3	5,8	
**4**	g TOTAL	2,9	5,7	8,6	14,3	20,0	25,7	34,3	42,9	
	g RET	2,0	2,4	2,9	2,3	3,2	**6,1 (23,7%)**	4,3	1,8	
	**Σ**	**8,9**	**7,8**	**9,6**	**7,5**	**11,3**	**19,7**	**12,4**	**9,5**	
**8**	g TOTAL	3,4	6,8	10,2	16,9	23,7	30,5	40,6	50,8	**KUN**
	g RET	1,1	1,5	2,4	1,4	3,4	**4,1 (13,5)**	2,7	1,8	
	**Σ**	**1,1**	**1,5**	**2,4**	**1,4**	**3,4**	**4,1**	**2,7**	**1,8**	
**10**	g TOTAL	0,5	1,0	1,5	2,5	3,5	4,5	6,0	7,5	
	g RET	0,5	0,8	1,0	1,4	2,2	**2,9 (66.6%)**	2,5	3,4	
**13**	g TOTAL	3,5	7,0	10,6	17,6	24,7	31,7	42,3	52,9	**PLA_2**
	g RET	3,5	6,5	10,3	14,6	15,7	**17,5 (55%)**	16,4	17,5	
	**Σ**	**3,5**	**6,5**	**10,3**	**14,6**	**15,7**	**17,5**	**16,4**	**17,5**	
**14**	g TOTAL	2,8	5,6	8,3	13,9	19,5	25,0	33,4	41,7	**Cystatin**
	g RET	2,8	5,6	8,3	12,1	12,9	**13,3 (53%)**	10,1	11,5	
	**Σ**	**2,8**	**5,6**	**8,3**	**12,1**	**12,9**	**13,3**	**10,1**	**11,5**	
**15**	g TOTAL	12,3	24,6	36,9	61,5	86,1	110,7	147,6	184,6	**SerPro**
	g RET	11,5	23,6	34,5	**39,6 (64.4%)**	38,7	38,6	37,5	11,9	

**16**		g TOTAL	2,0	4,0	6,0	10,0	14,1	18,1	24,1	30,1	
		g RET	2,0	4,0	6,0	9,0	9,4	**10,0 (55.2%)**	9,6	7,5	
**18**		g TOTAL	6,0	11,9	17,9	29,8	41,7	53,6	71,4	89,3	
		g RET	6,0	11,9	17,9	25,9	29,5	**31,2 (59%)**	29,0	20,4	
	**Σ**		**19,5**	**39,5**	**58,4**	**74,5**	**77,6**	**79,8**	**76,1**	**39,8**	
**20**		g TOTAL	4,3	8,7	13,0	21,7	30,4	39,1	52,2	65,2	
		g RET	4,3	8,5	12,9	20,3	27,8	32,0	**34,6 (66.3)**	31,0	
**22**		g TOTAL	13,5	27,0	40,5	67,5	94,5	121,5	161,9	202,4	**CTL**
		g RET	13,4	26,9	40,1	61,3	64,3	**70,6 (58,1%)**	66,3	43,8	
**25**		g TOTAL	1,4	2,8	4,2	7,0	9,8	12,6	16,8	21,0	
		g RET	1,4	2,8	4,0	6,2	8,6	10,7	**13,1 /78%)**	13,0	
	**Σ**		**19,1**	**38,2**	**57,0**	**86,8**	**100,7**	**113,3**	**114,0**	**87,8**	
**26**		g TOTAL	26,8	53,5	80,3	133,8	187,3	240,8	321,1	401,4	
		g RET	26,3	48,9	**55,5 (69,1%)**	42,8	37,4	34,5	24,9	14,1	**PI-SVMP**
	**Σ**		**26,3**	**48,9**	**55,5**	**42,8**	**37,4**	**34,5**	**24,9**	**14,1**	
**28**		g TOTAL	1,8	3,5	5,3	8,8	12,3	15,8	21,1	26,3	
		g RET	1,8	3,5	5,3	8,8	12,3	15,8	**18,2 (86,3%)**	18,2	**PIII-SVMP**
**29**		g TOTAL	1,6	3,2	4,8	7,9	11,1	14,3	19,0	23,8	
		g RET	1,4	3,2	4,8	7,9	11,1	14,3	**17,7 (93,2%)**	16,8	
	**Σ**		**3,2**	**6,7**	**10,1**	**16,7**	**23,4**	**30,1**	**35,9**	**35,0**	

Maximal binding capacity: 348,1 g venom proteins retained by 8 mg EchiTAb-Plus-ICP (= 43,5 mg venom proteins/g IgG)

Maximal binding for each RP-HPLC fraction is highlighted in yellow background. Green background, amount (in g) of toxin family proteins immunoretained in the affinity columns.

Table S2. Total and immunoretained (RET) *N. melanoleuca* (Ghana) venom proteins by EchiTAb-Plus-ICP® antivenom affinity column.

RP-HPLC fraction		100	200	300	500	700	900	1200	Toxin class
		***Naja melanoleuca* (Ghana) total venom proteins g)**							
**1**	g TOTAL	0,3	0,7	1,0	1,7	2,3	3,0	4,0	
	g RET	0,0	0,0	0,0	0,0	0,0	0,0	0,0	
**3**	g TOTAL	12,6	25,3	37,9	63,2	88,5	113,8	151,8	
	g RET	URQ*	URQ*	URQ*	URQ*	URQ*	URQ*	URQ*	
**4**	g TOTAL	3,3	6,7	10,0	16,6	23,3	29,9	39,9	
	g RET	0,0	0,1	0,0	0,0	0,0	0,0	0,0	
**6**	g TOTAL	0,6	1,2	1,9	3,1	4,3	5,6	7,4	
	g RET	0,0	0,0	0,0	0,0	0,0	0,0	0,0	
**7**	g TOTAL	2,4	4,8	7,2	12,0	16,8	21,6	28,8	
	g RET	URQ*	URQ*	URQ*	URQ*	URQ*	URQ*	URQ*	
**8**	g TOTAL	10,8	21,6	32,3	53,9	75,4	97,0	129,3	**3FTx (8-14 min)**
	g RET	URQ*	URQ*	URQ*	URQ*	URQ*	URQ*	URQ*	
**9**	g TOTAL	6,6	13,2	19,8	33,0	46,3	59,5	79,3	
	g RET	URQ*	URQ*	URQ*	URQ*	URQ*	URQ*	URQ*	
**10**	g TOTAL	0,5	1,0	1,5	2,5	3,5	4,5	6,0	
	g RET	NRQ	NRQ	NRQ	NRQ	NRQ	NRQ	NRQ	
**11**	g TOTAL	1,0	2,1	3,1	5,1	7,2	9,2	12,3	
	g RET	URQ*	URQ*	URQ*	URQ*	URQ*	URQ*	URQ*	
**12**	g TOTAL	1,1	2,2	3,3	5,5	7,7	9,9	13,2	
	g RET	URQ*	URQ*	URQ*	URQ*	URQ*	URQ*	URQ*	
**13**	g TOTAL	0,7	1,3	2,0	3,4	4,7	6,0	8,1	
	g RET	URQ*	URQ*	URQ*	URQ*	URQ*	URQ*	URQ*	
	**Σ**	**0,0**	**0,0**	**0,0**	**0,0**	**0,0**	**0,0**	**0,0**	
**15**	g TOTAL	0,4	0,9	1,3	2,2	3,1	4,0	5,3	
	g RET	0,4	0,8	1,1	1,8	2,2	**2,5 (62,5%)**	2,3	**PLA_2**
**16**	g TOTAL	10,3	20,6	30,9	51,5	72,2	92,8	123,7	

		g RET	9,8	10,8	**11,5 (37,2%)**	10,7	10,4	8,0	1,0	
**17**		g TOTAL	0,2	0,4	0,6	0,9	1,3	1,7	2,2	
		g RET	0,2	0,3	0,4	0,5	**0,7 (53,8%)**	0,5	0,6	
**18**		g TOTAL	2,3	4,7	7,0	11,7	16,4	21,0	28,0	
		g RET	2,3	4,2	5,3	5,0	5,0	**5,8 (27,6%)**	5,5	
**20**		g TOTAL	4,9	9,7	14,6	24,3	34,0	43,7	58,2	
		g RET	4,6	6,6	**7,2 (49,3%)**	5,8	5,7	6,1	6,1	
**21**		g TOTAL	4,4	8,7	13,1	21,8	30,5	39,2	52,3	
		g RET	3,3	4,9	7,8	4,1	11,5	20,1	**24,2 (45,9%)**	
	**Σ**		**20,9**	**27,6**	**33,3**	**27.9**	**35,5**	**43,0**	**41,2**	
**22**		g TOTAL	12,0	24,0	36,1	60,1	84,2	108,2	144,3	
		g RET	9,8	12,0	12,6	12,8	**24 (28,5%)**	14,3	1,2	
**23**		g TOTAL	1,5	3,1	4,6	7,7	10,8	13,9	18,6	**3FTx (18-22 min)**
		g RET	1,5	2,2	2,8	2,5	2,8	**3,7 (26,6%)**	2,7	
**24**		g TOTAL	0,2	0,4	0,6	1,1	1,5	1,9	2,6	
		g RET	URQ*	URQ*	URQ*	URQ*	URQ*	URQ*	URQ*	
	**Σ**		**11,5**	**14,6**	**16,0**	**16,4**	**28,3**	**19,9**	**6,2**	
**27**		g TOTAL	0,6	1,3	1,9	3,2	4,5	5,8	7,8	**Vespryn**
		g RET	-0,2	0,4	0,4	0,4	0,2	**0,8 (13,8%)**	0,0	
	**Σ**		**-0,2**	**0,4**	**0,4**	**0,4**	**0,2**	**0,8**	**0,0**	
**29**		g TOTAL	3,9	7,8	11,6	19,4	27,2	34,9	46,6	
		g RET	3,5	5,3	**6,5 (56%)**	4,8	5,1	5,3	0,3	
**30**		g TOTAL	0,9	1,8	2,7	4,6	6,4	8,2	11,0	**CRISP**
		g RET	0,7	0,7	**0,9 (33,3%)**	0,5	0,9	0,7	0,8	
**32**		g TOTAL	4,7	9,3	14,0	23,3	32,6	41,9	55,9	
		g RET	3,4	5,5	7,2	8,2	10,1	**10,7 (25,5%)**	5,7	
	**Σ**		**7,6**	**11,5**	**14,6**	**13,5**	**16,1**	**16,7**	**6,8**	
**34**		g TOTAL	1,1	2,1	3,2	5,3	7,5	9,6	12,8	
		g RET	1,1	2,1	3,2	**3,6 (67,9%)**	2,8	2,4	3,1	**PIII-SVMP**
**35**		g TOTAL	6,0	12,0	18,0	30,0	42,0	54,0	72,0	

	g RET	6,0	10,5	14,0	14,8	15,8	**16,5 (30,6%)**	7,3	
**Σ**		**7,6**	**13,5**	**18,6**	**19,7**	**19,5**	**20,2**	**12,0**	
**36**	g TOTAL	0,3	0,6	0,9	1,5	2,1	2,8	2,7	**PDE**
	g RET	0,3	0,6	0,9	1,5	2,1	**2,8 (100%)**	2,7	
**Σ**		**0,3**	**0,6**	**0,9**	**1,5**	**2,1**	**2,8**	**2,7**	
**37**	g TOTAL	1,6	3,2	4,7	7,9	11,0	14,2	18,9	
	g RET	1,2	2,7	3,5	6,7	7,9	**8,7 (61,2%)**	6,1	
**38**	g TOTAL	0,6	1,1	1,7	2,8	3,9	5,0	6,7	**LAO**
	g RET	0,6	1,1	1,5	2,4	**2,8 (72%)**	2,6	2,5	
**39**	g TOTAL	3,6	7,3	10,9	18,2	25,5	32,8	43,8	
	g RET	3,5	7,1	10,7	16,9	20,7	**23,8 (72,5%)**	23,8	
**Σ**		**5,3**	**10,9**	**15,7**	**26,0**	**31,4**	**35,1**	**32,4**	

Maximal binding capacity: 156,5 g venom proteins retained by 8 mg EchiTAb-Plus-ICP (= 19,6 mg venom proteins/g IgG)

Maximal binding for each RP-HPLC fraction is highlighted in yellow background. Green background, amount (in g) of toxin family proteins immunoretained in the affinity columns. * URQ, Unreliable quantification; indistinguishable from non-specific binding background.
